# Stapler-induced vascular injury during uniportal VATS lobectomy: lessons learned from a rare complication case

**DOI:** 10.1186/s40792-024-02048-9

**Published:** 2024-10-28

**Authors:** Yasuhiro Nakashima, Mariko Hanafusa, Hironori Ishibashi, Hiroshi Hosoda

**Affiliations:** 1 Department of Thoracic Surgery, Tokyo Kyosai Hospital, 2-3-8 Nakameguro, Meguro-ku, Tokyo, 153-8934 Japan; 2 Department of Thoracic Surgery, Institute of Science Tokyo, 1-5-45 Yushima, Bunkyo-ku, Tokyo, 113-8519 Japan; 3 Division of Cohort Research, National Cancer Center Institute for Cancer Control, Tokyo, 104-0045 Japan; 4 Department of Respiratory Medicine, Hokuetsu Hospital, Midori-cho, Shibata-shi, Niigata, 957-0018 Japan

**Keywords:** Uniportal video-assisted thoracoscopic surgery (VATS), Pulmonary artery injury, Staple stumps, Intraoperative complications, Thoracoscopic vascular clamping, Lung cancer

## Abstract

**Background:**

Due to advances in video-assisted thoracic surgery (VATS), the majority of lung resections can be performed safely via VATS with low morbidity and mortality. However, pulmonary artery (PA) bleeding often requires emergency conversion to thoracotomy, potentially leading to a life-threatening situation. We report a case of pulmonary artery injury caused by an unexpected stapler-tissue interaction during uniportal VATS lobectomy, highlighting the importance of recognizing and managing such rare complications to improve patient outcomes.

**Case presentation:**

A 63-year-old man underwent uniportal VATS left upper lobectomy for a suspected primary lung cancer. During the procedure, unexpected bleeding occurred from the third branch of the pulmonary artery (A3) after withdrawal of an unfired stapler. The protruding staple of the A3 stump was inadvertently hooked and stretched by the groove of the staple anvil. Although the bleeding was controlled by compression with the lung, the injured A3 stump required repair. Due to the extensive intimal injury near the central part of the left main pulmonary artery and the potential risk of fatal postoperative complications, we converted to open thoracotomy for definitive vascular repair by suturing. The patient had no postoperative complications and was discharged on postoperative day 8.

**Conclusions:**

This case report provides valuable lessons regarding the rare stapler-related vascular injury during uniportal VATS lobectomy. It is important to note that even during non-vascular dissection, unexpected stapler-tissue interactions can lead to bleeding. To prevent the vessel stump entanglement with stapler components, maintaining separation between the stapler and staple stumps is crucial. In uniportal VATS, manipulation during stapler insertion is one of the most challenging phases for instrument interference, requiring increased caution to prevent complications such as the vascular injury described in this case. Thorough preoperative planning, specific intraoperative precautions, and adapted safety protocols that address the limitations of uniportal VATS are essential for effective management of potential complications. Although techniques for thoracoscopic vascular control exist, they are not always feasible and conversion to open thoracotomy should be considered when necessary to ensure patient safety.

**Supplementary Information:**

The online version contains supplementary material available at 10.1186/s40792-024-02048-9.

## Background

Video-assisted thoracoscopic surgery (VATS) offers many advantages over traditional open thoracic surgery and is widely accepted for a variety of thoracic procedures. Single-port VATS, one of the developments in VATS, is an approach that has gained acceptance in the treatment of lung lesions since it was first reported in 2011 [1]. Despite the development of thoracoscopic surgical approaches, the most common intraoperative complication that surgeons are concerned about is bleeding, and in fact this bleeding is the most common reason for emergency conversion to thoracotomy during the learning curve. The reported incidence of vascular injury during VATS lobectomy ranges from 2.2% to 4.1% [2]. In these cases, patients often require hemostatic procedures with angiorrhaphy, with or without a sealant, due to injuries to the pulmonary artery and vein [3]. The majority of these injuries are caused by technical problems related to the use of energy devices, staplers, or forceps, such as rough maneuver, thermal injury, or visibility issues [3]. Serious bleeding resulting from these injuries is considered the most troublesome and dangerous condition, leading to emergency conversion to thoracotomy [4].

To minimize the risk of major bleeding during VATS, it is essential for surgeons to be aware of rare vascular injury patterns. Furthermore, effective methods to manage controllable vascular injuries without emergency conversion to thoracotomy are rarely reported in the literature [4]. Developing and sharing techniques for repairing injured vessels through VATS could help reduce the need for emergency thoracotomy and improve patient outcomes.

Here, we present a case of pulmonary artery injury caused by a rare stapler-induced complication during uniportal VATS lobectomy. This case report aims to share our experience and lessons learned from this uncommon complication, emphasizing the significance of preventive measures and the need for effective management strategies when dealing with stapler-induced vascular injury during VATS procedures.

## Case presentation

A 63-year-old man was referred to our hospital after a chest computed tomography (CT) scan during a routine health check-up incidentally revealed a nodule in the left upper lobe of the lung.

The patient's medical history included bladder cancer, which was surgically treated at the age of 63, and hypertension, which was being managed with medication. He had a smoking history of 84 pack-years but had quit smoking 2 months before the surgery. After the initial presentation, the left lung nodule showed a tendency to grow over time. Therefore, surgery was planned for diagnostic and therapeutic purposes. Physical examination did not reveal any significant abnormalities. Laboratory tests showed a slightly elevated CYFRA level of 2.43 ng/mL, but no other notable tumor marker elevations were observed. Spirometry revealed a slight decrease in the forced expiratory volume in one second ratio.

Chest CT demonstrated emphysematous changes predominantly in the upper lobes of the lungs (Video S1). The tumor, measuring 18 mm in diameter, was a solid lesion located within the wall of a cyst adjacent to the interlobar fissure in the left upper lobe (S1 + 2c). Magnetic resonance imaging with diffusion-weighted imaging with background suppression revealed a region of reduced diffusion corresponding to the left upper lobe lung nodule. No findings suggestive of distant metastases to other organs were observed.

The clinical diagnosis was primary lung cancer (cT1bN0M0, cStageIA2) without definitive pathological confirmation. A thoracoscopic (uniportal) left upper lobectomy and lymph node dissection under general anesthesia was planned for both diagnostic and therapeutic purposes. A 4-cm incision was made in the sixth intercostal space along the mid-axillary line.

**Fig. 1:**
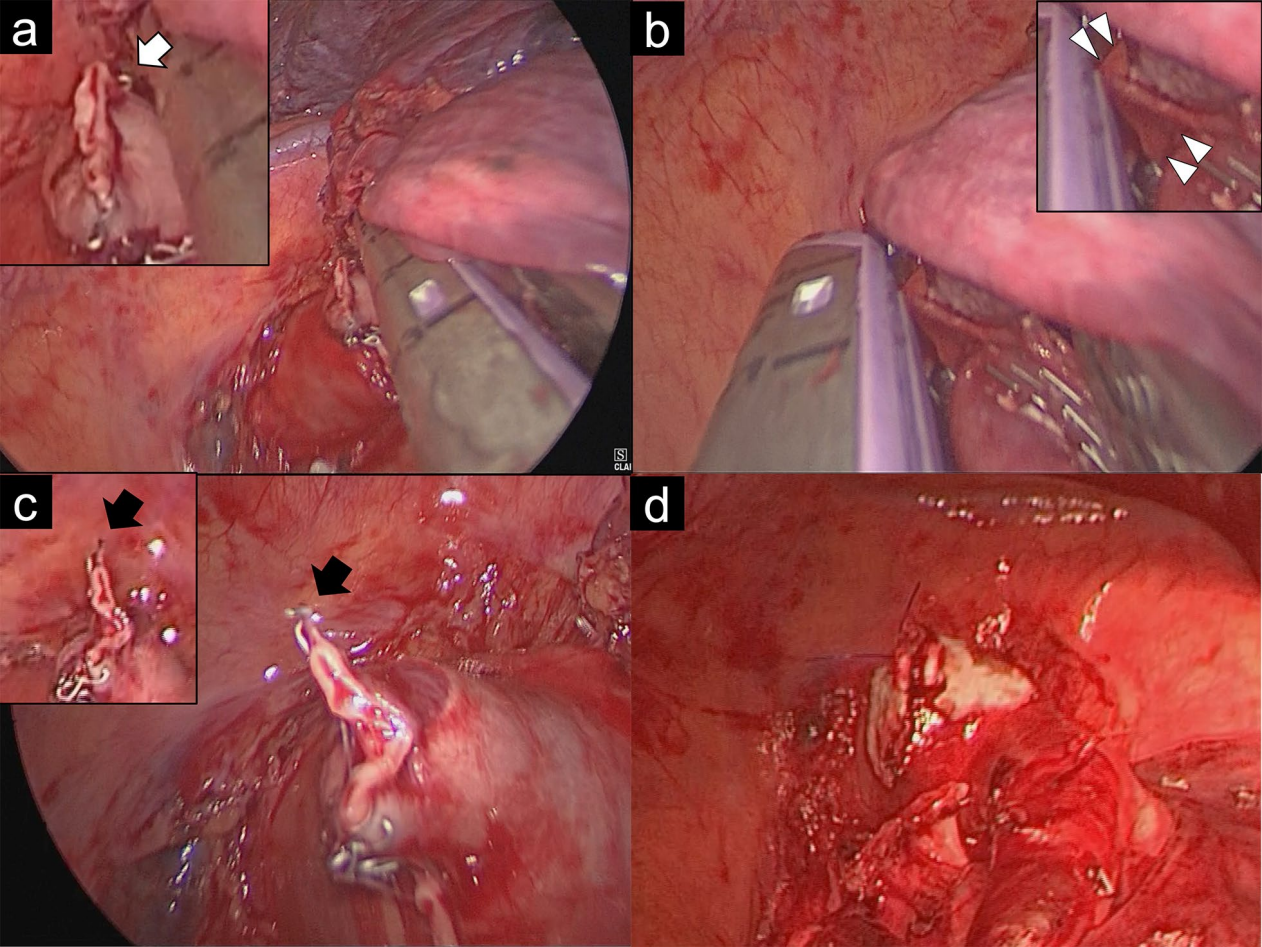
Vascular injury with staple and repair: Intraoperative images. **a** Pre-injury state: The A3 stump showed no injury and part of the staple protruded toward the head (white arrow). **b** Moment of injury: The A3 stump is hooked and stretched toward the back of the stapler anvil (white triangle). The stapler is being withdrawn, creating tension on the vessel. **c** Post-injury state: Intimal injury at the proximal end of the A3 is evident. The previously protruding staple is now deformed (black arrow), consistent with being caught and stretched. **d** Repair: The A3 injury site was reinforced with a *U*-suture using a 4-0 Asflex suture with a pledget and application of a piece of sealant

Intraoperative frozen sections of core needle biopsy of the tumor protruding from the interlobar surface of the upper lobe revealed squamous cell carcinoma. The upper pulmonary vein and branches of the upper lobe pulmonary artery were divided using a Signia™ Small Diameter Reload Short with Curved Tip, 30 mm, white stapler (Medtronic, Minneapolis, MN, USA). No vascular injury was observed during the process of dividing the vessels (Fig. 1a).

**Fig. 2:**
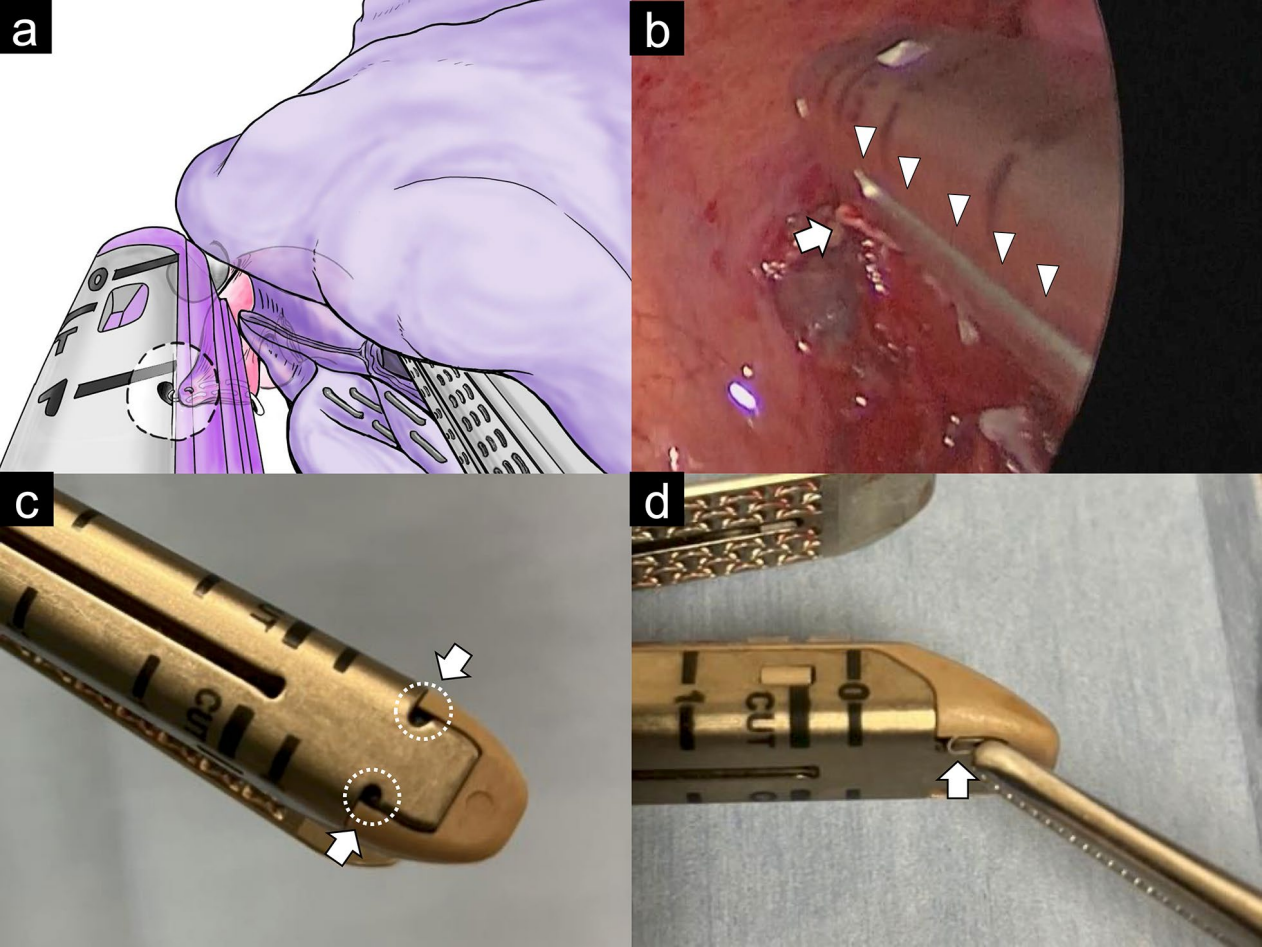
Mechanism of stapler-induced pulmonary artery injury during uniportal VATS lobectomy. **a** Illustration based on Fig. 1b: A deformed staple from the A3 stump is caught in a small hole of the stapler's clamp cover, stretching the A3 stump. This interaction was not visible during the procedure due to its position behind the staple line and the interlobar tissue. **b** Moment just after opening the stapler, before stretching the vessel: the vessel stump has not yet entered the I-shaped groove of the external cap on the *I*-beam-like component of the stapler reload, as reported in previous cases. **c** Close-up view of the COVIDIEN Tri-Staple 2.0 stapler cover, showing the small hole (white arrow) where the protruding stump staple became entangled in this case. **d** Simulation demonstrating how a Tri-Staple 2.0 staple (white arrow) can potentially become trapped in the small hole of the stapler cover

Next, the anterior interlobar fissure was partially divided using a Signia™ Tri-Staple 2.0 Reinforced Intelligent Reload, 45 mm, Black (Medtronic, Minneapolis, MN, USA). To divide the remaining the fissure, a Signia™ Tri-Staple 2.0 Reinforced Intelligent Reload, 45 mm, Purple (Medtronic, Minneapolis, MN, USA) was inserted and clamped. While attempting to confirm that the staples were clear of the pulmonary artery by looking over the staples to view the hilar tissue behind them, the clamped thin lung tissue shifted slightly distally to the staples. Limited maneuverability with the stapler inserted made it difficult to reposition the displaced lung tissue. It was decided to temporarily withdraw the stapler in order to reposition the lung tissue. Upon opening the unfired stapler for withdrawal, an unexpected resistance was encountered. The hilar vessels being pulled behind the lung fissure and the staples were not recognized (Fig. 1b). The resistance abruptly ceased, and bleeding from the pulmonary hilum was observed immediately after complete withdrawal of the stapler. After the bleeding was controlled by compression with the upper lobe, an intimal injury was found on the proximal end of the stump of the third branch of the pulmonary artery (A3), and the protruding staple was deformed (Fig. 1c, Video S2). The mechanism of vascular injury was revealed that the protruding staple on the edge of the A3 stump was hooked in a groove on the back of the anvil of the stapler, causing vascular injury when the stapler was withdrawn (Fig. 2). As hemostasis was achieved, the remaining anterior interlobar fissure and upper lobe bronchus were divided using staplers, and the left upper lobe was removed. Intraoperative inspection revealed that the intimal injury was extensive, involving more than half of the thick A3 stump. The damaged area was located near the central part of the left main pulmonary artery, a site prone to tension. Considering the extent and location of the injury, we determined that any subsequent bleeding could be potentially fatal and that reinforcement with sealants alone would be insufficient to prevent potential postoperative complications. Based on this assessment, we proceeded with a definitive vascular repair by suturing. Although we attempted to reinforce the injured A3 stump thoracoscopically using side-clamping or endoloop ligation, it was difficult to clamp the proximal pulmonary artery thoracoscopically. Therefore, the procedure was converted to open thoracotomy, extending the incision to 13 cm, and the left main pulmonary artery was clamped with a tourniquet. The A3 injury site was reinforced with a *U*-suture using a 4-0 Asflex suture with a pledget and application of a piece of sealant (cut 1 cm × 1 cm) TachoSil^®^ (Nycomed GmbH, Linz, Austria) (Fig. 1d). The operative time was 412 minutes with a blood loss of 50 mL.

The patient had an uneventful postoperative course and was discharged on postoperative day 8. The pathological diagnosis was squamous cell carcinoma of the left upper lung (pT1cN0M0, pStageIA3). The patient was followed up in the outpatient clinic without any problems.

## Discussion

This case report provides instructive insights into vascular injury during uniportal VATS lobectomy. We learned that vessel stump with protruding staple can be hooked into a groove on the back of the stapler anvil, potentially causing vascular injury. Additionally, we realized that thorough preparation and team simulation for thoracoscopic clamping of the proximal main pulmonary artery are essential for managing injuries such as the left A3 pulmonary artery injury encountered in this case.

We had not anticipated that an open stapler could potentially pull and damage tissue in this way. In a previously reported case [5], vascular injury was avoided because the entanglement of deformed vessel stump staples with the external cap on the I-beam-like component of the stapler reload was visible. In our case, the mechanism was slightly different. The protruding vascular stump staples became entangled in a small hole on the stapler cover located in a blind spot, resulting in vessel injury (Fig. 2a-b). This rare risk of complication is more likely to occur with designs that have a groove, such as those made by Covidien. Staple designs from other major manufacturers without a groove in the staple cap might have a lower risk of similar complications [5]. However, some surgeons who value the safety of a gentler closure that doesn't compress the entire clamped tissue when the stapler closes (as seen in other manufacturers) might consider this unusual safety concern acceptable [6]. Our case further emphasizes the need for surgeons to be aware of this potential complication and to avoid bringing the underside of the stapler and the staple stumps close to each other to prevent such mishaps. In addition, if any unexpected resistance is encountered during stapler manipulation, it is essential to thoroughly examine the groove on the back of the stapler anvil for potential tissue entrapment, regardless of whether the stapler is open or closed. This case is instructive not only for careful staple manipulation, but also for the need to monitor for misplaced, partially embedded, or malformed staples during surgery. This case highlights the critical importance of not only careful staple handling, but also vigilant intraoperative monitoring for errant, partially embedded, and malformed staples. For small vessel division, where there is less vascular wall and a higher risk of staple protrusion, alternative methods such as energy devices should be considered [6].

In our case, although bleeding was controlled by lung compression, we determined that the use of sealants alone would be insufficient due to the extensive intimal damage to the vessel. The use of sealants should be reserved for situations where the defect is small or where control by means of suture or ligation is complex [7]. Furthermore, a case of critical bleeding from a tear near the distal end of the stump of the first branch of the left main pulmonary artery has been reported, despite no bleeding problems occurring during the surgery [8]. The bleeding occurred while the patient was awakening from general anesthesia, likely due to tension on the central part of the pulmonary artery stump. The location of the vascular injury in our case was similar, which further supported our decision to repair the damage with vascular suturing.

Several techniques have been proposed for thoracoscopic clamping of the pulmonary artery [4, 9, 10], but these techniques have not been widely adopted. In our case, the location of the injury on the left A3 made thoracoscopic clamping particularly challenging [4], necessitating conversion to open thoracotomy. This decision aligns with the principle that patient safety should always take precedence over completing the procedure thoracoscopically [3].

While the uniportal VATS approach offers benefits such as reduced postoperative pain and faster recovery, it also presents unique challenges that may have contributed to this complication [7]. The limited visibility and instrument maneuverability inherent in the uniportal technique can make it more difficult to identify and avoid potential tissue-instrument interactions. Unlike other uniportal VATS-specific instruments with thin shafts, the larger diameter stapler significantly reduces the available space at the incision site, further limiting the range of motion for instruments. In this case, attempting to inspect the posterior aspect of the stapler after its insertion for fissure division brought it closer to the vessel stumps in the hilum. This safety check, routinely performed in multiport VATS, may need to be reconsidered in the uniportal approach due to its limitations. When dividing the fissure, it is crucial to secure the target tissue away from vital structures prior to stapler insertion, eliminating the need for posterior inspection. Alternatively, careful reinsertion of the rigid scope from behind the stapler without excessive movement is essential.

This case report provides valuable insight into the recognition and management of rare complications during uniportal VATS lobectomy, with important implications for clinical practice and future research. Recognizing the potential for stapler-related vascular injury, understanding the mechanisms behind such complications, and developing strategies to prevent and manage these events are crucial for improving the safety and efficacy of minimally invasive thoracic surgery. Furthermore, this case highlights the need for thorough preparation and team simulation when dealing with intraoperative complications. Future studies should focus on developing and validating standardized protocols for managing vascular injury during VATS procedures and investigating the effectiveness of various techniques for thoracoscopic vascular control.

## Conclusions

Stapler-related vascular injury is a rare but potentially serious complication during uniportal VATS lobectomy. Surgeons should be aware of the risk of staple stumps becoming entangled in stapler components and take precautions to avoid such mishaps. Thorough preparation and team simulation are essential for effective managevent of intraoperative complications, particularly when dealing with vascular injuries. While techniques for thoracoscopic vascular control exist, they may not always be feasible or practical, and conversion to open thoracotomy should be considered when necessary to ensure patient safety and optimal outcomes.

## Supplementary Information

Additional file 1.

Additional file 2.

## Data Availability

The data supporting the conclusions of this article are included within the article.

## Abbreviations

VATS: Video-assisted thoracic surgery
A3: The third branch of the pulmonary artery
CT: Computed tomography

## References

[1] Gonzalez D, Paradela M, Garcia J, Dela TM. Single-port video-assisted thoracoscopic lobectomy. Interact Cardiovasc Thorac Surg. 2011;12(3):514–5. 10.1510/icvts.2010.256222. 21131682 10.1510/icvts.2010.256222

[2] Subotic D, Hojski A, Wiese M, Lardinois D. Use of staplers and adverse events in thoracic surgery. J Thorac Dis. 2019;11(Suppl 9):S1216–21. 10.21037/jtd.2019.03.13. 31245090 10.21037/jtd.2019.03.13 PMC6560572

[3] Miyazaki T, Yamasaki N, Tsuchiya T, Matsumoto K, Hatachi G, Kitamura Y, et al. Management of unexpected intraoperative bleeding during thoracoscopic pulmonary resection: a single institutional experience. Surg Today. 2016;46(8):901–7. 10.1007/s00595-015-1253-9. 26411432 10.1007/s00595-015-1253-9

[4] Mei J, Pu Q, Liao H, Ma L, Zhu Y, Liu L. A novel method for troubleshooting vascular injury during anatomic thoracoscopic pulmonary resection without conversion to thoracotomy. Surg Endosc. 2013;27(2):530–7. 10.1007/s00464-012-2475-1. 22806532 10.1007/s00464-012-2475-1 PMC3580039

[5] Matsui H, Murakawa T. Potential surgical challenge: hooking the staple stump. JTCVS Tech. 2021;11:76–7. 10.1016/j.xjtc.2021.10.051. 35169745 10.1016/j.xjtc.2021.10.051 PMC8828788

[6] Demmy TL. Commentary: How did that happen? JTCVS Tech. 2021;11:78–9. 10.1016/j.xjtc.2021.11.013. 35169746 10.1016/j.xjtc.2021.11.013 PMC8828979

[7] Gonzalez-Rivas D, Stupnik T, Fernandez R, de la Torre M, Velasco C, Yang Y, et al. Intraoperative bleeding control by uniportal video-assisted thoracoscopic surgery. Eur J Cardiothorac Surg. 2016;49(Suppl 1):i17–24. 10.1093/ejcts/ezv333. 26424873 10.1093/ejcts/ezv333

[8] Kamiyoshihara M, Igai H, Matsuura N, Yazawa T, Ohsawa F, Furusawa S. Critical bleeding from the stapled stump of the pulmonary artery. Kitakanto Med J. 2021;71(2):143–5. 10.2974/kmj.71.143.

[9] Kamiyoshihara M, Nagashima T, Ibe T, Takeyoshi I. A tip for controlling the main pulmonary artery during video-assisted thoracic major pulmonary resection: the outside-field vascular clamping technique. Interact Cardiovasc Thorac Surg. 2010;11(5):693–5. 10.1510/icvts.2010.246132. 20724430 10.1510/icvts.2010.246132

[10] Watanabe A, Koyanagi T, Nakashima S, Higami T. How to clamp the main pulmonary artery during video-assisted thoracoscopic surgery lobectomy. Eur J Cardiothorac Surg. 2007;31(1):129–31. 10.1016/j.ejcts.2006.10.017. 17137789 10.1016/j.ejcts.2006.10.017

